# Adjuvant Injections Altered the Ileal and Fecal Microbiota Differently with Changes in Immunoglobulin Isotypes and Antimycobacterial Antibody Responses

**DOI:** 10.3390/ijms24032818

**Published:** 2023-02-01

**Authors:** Sundar Khadka, Seiichi Omura, Fumitaka Sato, Ikuo Tsunoda

**Affiliations:** Department of Microbiology, Kindai University Faculty of Medicine, Osaka 589-8511, Japan

**Keywords:** adjuvant, animal model, antibody isotype, bioinformatics, experimental autoimmune encephalomyelitis, *Mycobacterium tuberculosis*, pattern matching

## Abstract

Alterations in the gut microbiota, “dysbiosis,” have been reported in autoimmune diseases, including multiple sclerosis (MS), and their animal models. Although the animal models were induced by injections of autoantigens with adjuvants, including complete Freund’s adjuvant (CFA) and pertussis toxin (PT), the effects of adjuvant injections on the microbiota are largely unknown. We aimed to clarify whether adjuvant injections could affect the microbiota in the ileum and feces. Using 16S rRNA sequencing, we found decreased alpha diversities of the gut microbiota in mice injected with CFA and PT, compared with naïve mice. Overall, microbial profiles visualized by principal component analysis demonstrated dysbiosis in feces, but not in the ileum, of adjuvant-injected mice, where the genera *Lachnospiraceae NK4A136* group and *Alistipes* contributed to dysbiosis. When we compared the relative abundances of individual bacteria, we found changes in 16 bacterial genera in feces and seven genera in the ileum of adjuvant-injected mice, in which increased serum levels of antibody against mycobacteria (a component of CFA) and total IgG2c were correlated with the genus *Facklamia*. On the other hand, increased IgG1 and IgA concentrations were correlated with the genus *Atopostipes*. Therefore, adjuvant injections alone could alter the overall microbial profiles (i.e., microbiota) and individual bacterial abundances with altered antibody responses; dysbiosis in animal models could be partly due to adjuvant injections.

## 1. Introduction

Adjuvants are substances included in vaccines to enhance the immunogenicity of the antigens [1]. Adjuvants are required for antigens such as inactivated, subunit, and recombinant proteins, although adjuvants are not contained in live attenuated vaccines [2]. Complete Freund’s adjuvant (CFA) is the most commonly used adjuvant in experimental animals and consists of heat-killed *Mycobacterium tuberculosis* in incomplete Freund’s adjuvant (IFA). IFA is made from paraffin oil and mannide monooleate as a surfactant [3]. CFA has been used for the production of antigen-specific antibodies [4]; antigen/CFA emulsions have been extensively used in experimental immunology [5]. When CFA is mixed with an antigen, CFA forms a viscous water-in-oil emulsion with the antigen in the water phase [5]. Heat-killed *M. tuberculosis* in CFA contains components of the bacterial cell walls and unmethylated DNA, which can be recognized as pathogen-associated molecular patterns (PAMPs), activating the immune systems [3,5,6]. Although IFA does not carry PAMPs, IFA alone or in combination with antigens or other adjuvants has been shown to induce various immunomodulatory functions, including enhancement of antibody production and T cell subset polarization [7,8,9].

Multiple sclerosis (MS) is an immune-mediated disease in the central nervous system (CNS) [10]. Experimental autoimmune encephalomyelitis (EAE) has been used as an autoimmune model of MS and induced by sensitization with CNS antigens in animals [11]. In 1933, Rivers et al. induced EAE in monkeys with multiple intramuscular injections of rabbit brain emulsions/extracts without any adjuvants [12]. Later in 1947, Jules Freund induced EAE in guinea pigs successfully with a single injection of brain antigens with CFA [13]. CFA has also been most commonly used in the induction of other experimental models of autoimmune diseases: Guillain–Barré syndrome (GBS), myasthenia gravis (MG), myocarditis, orchitis, rheumatoid arthritis (RA), thyroiditis, and uveoretinitis [14,15,16,17,18,19,20,21,22,23,24,25] (Table 1). For example, experimental autoimmune neuritis (EAN), an animal model for GBS, can be induced by injection of peripheral myelin protein emulsified in CFA. Collagen-induced arthritis (CIA), an animal model for RA, can be induced by sensitizing with type II collagen emulsified in CFA. Mice sensitized with testicular homogenates (TH) emulsified in CFA developed more severe experimental autoimmune orchitis than mice sensitized with TH alone. Thus, in most autoimmune models, CFA has been required to induce full-blown clinical diseases [26], although sensitization with antigen alone was reported to induce more severe autoimmune diseases than that with CFA-emulsified antigen in a few models [27]. On the other hand, in several autoimmune models, injections of CFA-emulsified autoantigens have been shown to require additional adjuvants to induce diseases. Among adjuvants, pertussis toxin (PT) has been the most widely used additional adjuvant; PT injections are required for the induction of several autoimmune models, such as EAN, EAE, experimental autoimmune myocarditis (EAM), and experimental autoimmune uveoretinitis (EAU) [28] (Table 1). EAE has been induced by injecting various myelin antigens in several different animals. Although several EAE models can be induced by subcutaneous injection of CFA-emulsified myelin antigen alone, the most widely used EAE model in C57BL/6 mice requires sensitization of the myelin oligodendrocyte glycoprotein (MOG) peptide emulsified in CFA with additional PT injections.

Abundant and diverse microbial communities coexist in mammals, including humans and mice. In the gastrointestinal tract, the microbial communities are composed of microorganisms, including bacteria, archaea, fungi, and viruses, which are collectively referred to as the gut microbiota [29,30,31,32]. Since the microorganisms coexist in the gastrointestinal tract by regulating each other, changes of each microorganism could influence other microbial compositions, contributing to the pathogenesis of some diseases. Among the microorganisms, gut bacteria have been reported to exert either beneficial or pathogenic effects on host health conditions; alterations in gut microbiota compositions have been associated with the pathophysiology of a variety of disease conditions. For example, microbial diversities seem to play a role in maintaining health conditions; decreases in the diversities have been linked to various diseases, including Alzheimer’s disease [33] and inflammatory bowel disease (IBD) [34]. On the other hand, although there have been few reports that changes of fungi and viruses in the gastrointestinal tract could play either beneficial or detrimental roles in animal models, changes of the mycobiome and virome have been reported in some human diseases. For example, the altered mycobiome has been associated with bacterial dysbiosis in patients with IBD [35,36]. Bacteriophages in the gut play roles in bacterial existence and functions; the increased abundances of some phages have been reported in IBD patients [37,38].

In autoimmune diseases and their animal models, changes in the microbiota of feces have been proposed to play a key role in immune-mediated pathology [20,21,30,39]. For example, Heissigerova et al. demonstrated that decreases in bacterial loads prior to induction of EAU resulted in less severe inflammation [28]. Although most gut microbiota studies examined bacterial compositions using feces, the intestinal microbiota can be more crucial in the induction of several components of the immune systems, including immunoglobulin (Ig) A and T helper (Th) 17 cells than the fecal microbiota [40]. Previously, we reported that the ileal microbiota reflected disease activity, compared with the fecal microbiota in an EAE model [16]. Here, if the disease pathophysiology is associated with the intestinal microbiota, but not the fecal microbiota, the fecal microbiota changes would be irrelevant to clinical and immunological activities.

Although gut microbiota changes have been reported in several autoimmune animal models induced by injections of autoantigens emulsified in CFA, most reports compared the fecal microbiota of the model animals with that of naïve animals (Table 1). Only Johanson II et al. [15] reported changes in the gut microbiota of mice injected with CFA alone. They compared the fecal microbiota between EAE mice induced with MOG emulsified in CFA and PT injections versus the two controls: mice injected with CFA alone and naïve mice. CFA-injected mice had altered microbiota in feces, compared with naïve mice. When the relative abundances of bacteria were compared between CFA-injected and EAE mice, several bacterial families showed similar changes, compared with naïve mice. Although the overall microbiota profiles assessed by principal component analysis (PCA) in CFA-injected mice were more similar to those in naïve mice than EAE mice, CFA injection alone induced similar changes in certain individual bacterial abundances observed in EAE mice. Although Johanson II et al. did not describe whether the CFA-injection alone group received PT injections, their findings suggest that the gut microbiota changes reported in CFA-induced autoimmune models could be not only disease-associated, but also influenced by adjuvant injections themselves. If this is the case, the discrepancy seen in gut microbiota studies between human diseases versus animal models can be partly explained by the effects of CFA (with or without PT injections). Thus far, no study has examined whether adjuvant injections alone can alter bacterial compositions in the intestine.

In the current study, we aimed to determine whether adjuvant injections alone could affect the gut microbiota in the ileum and feces, following the most efficient adjuvant injections for immunomodulation, i.e., CFA with PT injections. We injected C57BL/6 mice with CFA and PT (“CFA-injected group”) and compared the gut microbiota with naïve mice (“naïve group”), and found significant differences in the alpha diversities in the fecal, but not ileal, microbiota. The overall microbiome profiles by PCA were different between the naïve and CFA-injected groups in feces, but not in the ileum. We also found changes in the relative abundances of individual bacteria in the CFA-injected group: 16 bacterial genera in feces and seven genera in the ileum. The CFA-injected group had higher levels of serum antimycobacterial and IgG2c antibodies, both of which were correlated with the relative abundance of the genus *Facklamia*. On the other hand, although the CFA-injected group had higher serum IgG1 and IgA antibodies, these isotype antibodies were correlated with different bacteria, i.e., the genus *Atopostipes*. Thus, we demonstrated that adjuvant injections alone could alter the overall microbiota profiles in feces and the relative abundances of individual bacteria, the latter of which was correlated with altered antibody responses.

## 2. Results

### 2.1. Decreased Alpha Diversities of the Fecal Microbiota in CFA-Injected Mice

**Figure 1:**
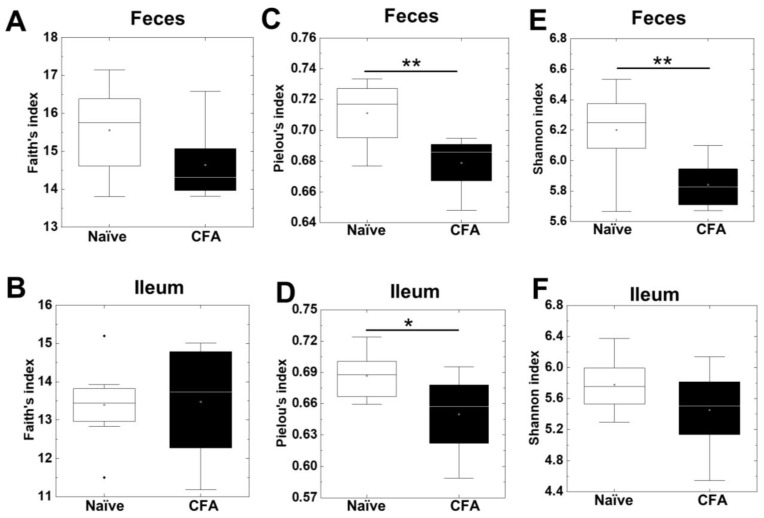
Alpha diversities of the microbiome in the ileum and feces were compared between naïve mice (white) and mice injected with complete Freund’s adjuvant (CFA) (black). The richness (number of genera), evenness, and combination of both were determined by the Faith’s phylogenetic diversity index (**A**,**B**), Pielou’s evenness index (**C**,**D**), and Shannon index (**E**,**F**), respectively. In feces, the Pielou’s evenness and Shannon indexes decreased significantly in the CFA-injected group (**, *p* < 0.01, Student’s *t* test). In the ileum, the Pielou’s evenness index decreased significantly in the CFA-injected group (*, *p* < 0.05, Student’s *t* test). In boxplots: the open circle, middle line, box, lower whiskers, upper whiskers, and dots indicate the mean, median, interquartile range, minimum, maximum and outliers, respectively. The total sample number was eight per group (naïve, n = 8; and CFA, n = 8).

To investigate whether CFA treatment could alter the diversities of the microbiota in the ileum and feces, we isolated bacterial DNA from the ileum and feces of naïve and CFA-injected mice. Using 16S rRNA sequencing, we determined the bacterial alpha diversities at the genus level by the Faith’s phylogenetic diversity, Pielou’s evenness, and Shannon indexes (Figure 1A–F). In the Faith’s indexes comparing the species richness (the number of bacterial genera), we did not find significant differences in the ileum or feces between the naïve and CFA-injected groups (Figure 1A,B). In the Pielou’s indexes comparing the evenness of the amounts of bacterial genera, the indexes of the ileal and fecal samples were significantly decreased in the CFA-injected group, compared with the naïve group (*p* < 0.05, Student’s *t* test, Figure 1C,D). In the Shannon indexes comparing the overall changes in diversities (combination of richness and evenness) between the two groups, we found decreases in the indexes of the CFA-injected group in feces, but not in the ileum (*p* < 0.01, Student’s *t* test, Figure 1E,F). Since microbiota compositions have been reported to differ among the sites of the gastrointestinal tract [41], we compared the alpha diversities of the microbiota between the ileum and feces. We found that all three indexes of fecal samples were significantly higher than those of the ileal samples in the naïve group, but not in the CFA-injected group (*p* < 0.05, Student’s *t* test, Supplemental Figure S1A–F).

### 2.2. Fecal, but Not Ileal, Microbiota Changes in CFA-Injected Mice

**Figure 2:**
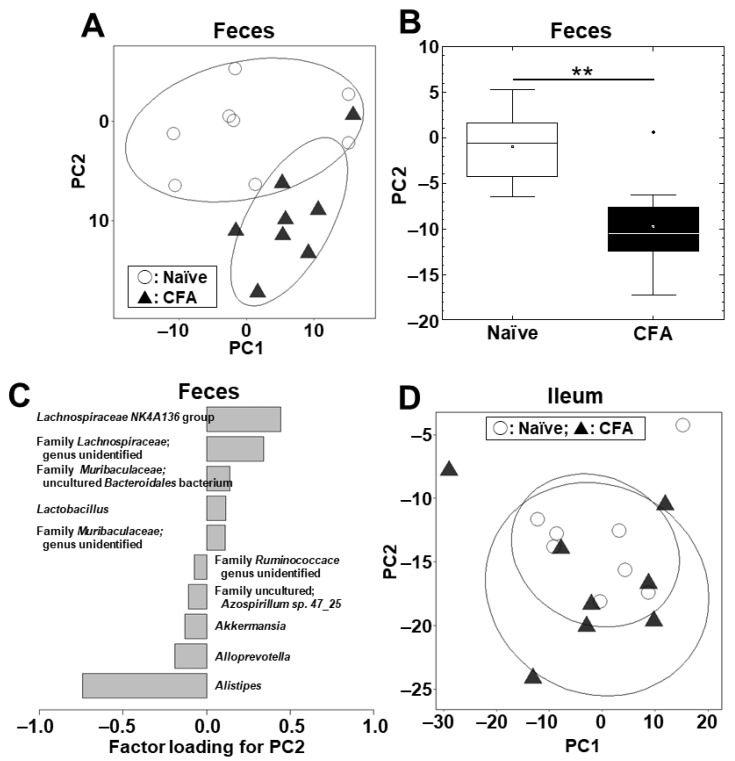
Principal component analysis (PCA) of the ileal and fecal microbiome data at the genus level between the naïve (○) and CFA-injected (▲) groups. We conducted PCA using the fecal (**A**–**C**) and ileal samples (**D**). Ellipses indicate an 80% confidence interval of each group. (**A**,**B**) In feces, PC2 values were statistically different between the two groups (**, *p* < 0.01, Student’s *t* test). (**C**) Factor loading for PC2 showed that the relative abundances of the genera *Lachnospiraceae NK4A136* group and *Alistipes* contributed positively and negatively to the PC2 distribution, respectively. (**D**) In the ileum, PCA did not separate the two groups. Each group was composed of eight mice.

We conducted PCA to compare the microbiome profiles in the ileal and fecal samples between the naïve and CFA-injected groups at the phylum (Supplemental Figure S2A) and genus (Figure 2A) levels. PCA separated the CFA-injected group from the naïve group in feces, but not in the ileum, by principal component (PC) 1 values at the phylum level (*p* < 0.01, Student’s *t* test, Supplemental Figure S2B) and by PC2 values at the genus level (*p* < 0.01, Student’s *t* test, Figure 2B). At the phylum level, factor loading for PC1 showed that the relative abundances of the phyla *Bacteroidetes* and *Firmicutes* contributed positively and negatively to the separation on the PC1 axis, respectively (Supplemental Figure S2C). At the genus level, factor loading for PC2 showed that the relative abundances of the genera *Lachnospiraceae NK4A136* group and *Alistipes* contributed positively and negatively to the separation on the PC2 axis, respectively (Figure 2C). In the ileum, neither PC1 nor PC2 values had statistical differences between the naïve and CFA-injected groups at the phylum (PC1, *p* = 0.59; and PC2, *p* = 0.22) and genus (PC1, *p* = 0.51; and PC2, *p* = 0.77) levels (Supplemental Figure S2D, Figure 2D).

### 2.3. Microbiota Differs between the Ileum and Feces in Naïve Mice, but Not in CFA-Injected Mice

**Figure 3:**
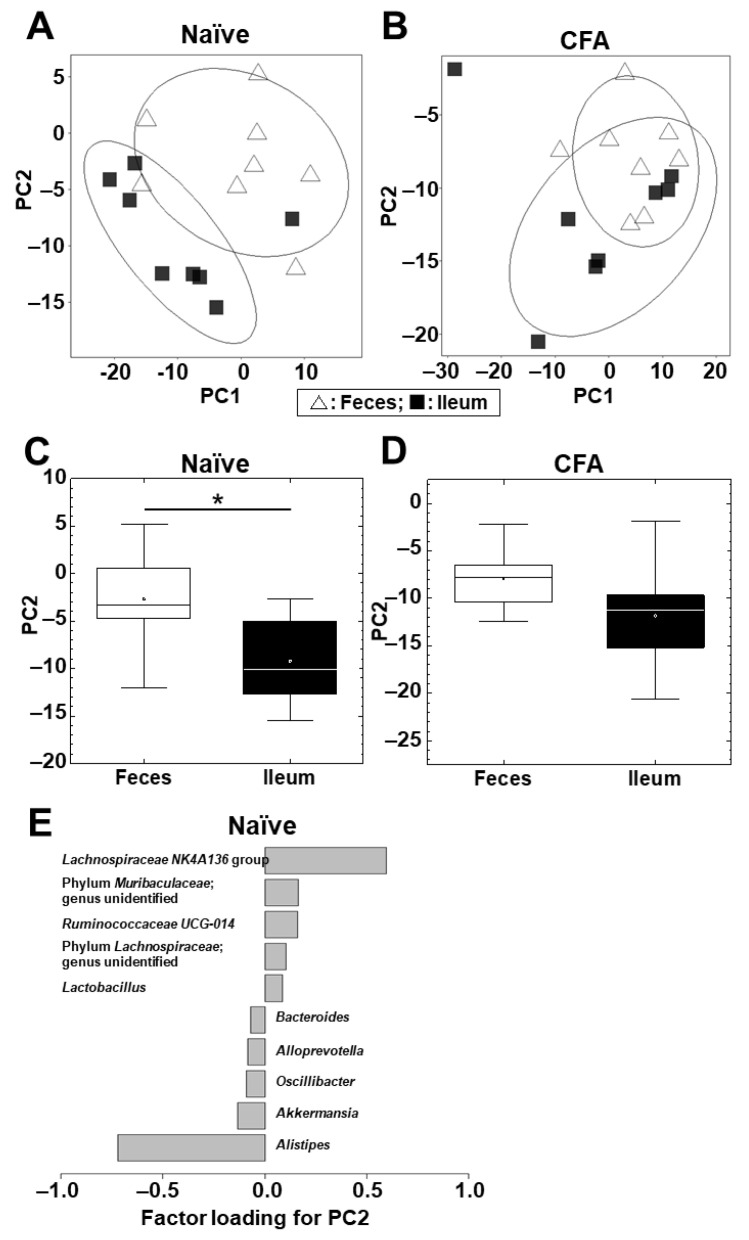
PCA of the microbiome data between the ileum (■) and feces (△) at the genus level in the naïve (**A**) and CFA-injected (**B**) groups. Ellipses indicate an 80% confidence interval of each group. PCA separated the ileum samples from the fecal samples by PC2 in the naïve group (**C**) (*, *p* < 0.05, Student’s *t* test), but not in the CFA-injected group (*p* = 0.11) (**D**). (**E**) Factor loading for PC2 showed that the relative abundances of the genera *Lachnospiraceae NK4A136* group and *Alistipes* contributed positively and negatively to the PC2 distribution, respectively, in the naïve group. Each group was composed of eight mice.

Next, we conducted PCA to compare the microbiome profiles between the ileum and feces in the naïve and CFA-injected groups (Supplemental Figure S3A,B; Figure 3A,B). In the naïve group, PCA separated the ileal microbiota from the fecal microbiota on the PC1 axis at the phylum level (*p* < 0.01, Student’s *t* test, Supplemental Figure S3C) and on the PC2 axis at the genus level (*p* < 0.05, Student’s *t* test, Figure 3C). At the phylum level, factor loading for PC1 showed that the relative abundances of the phyla *Bacteroidetes* and *Firmicutes* contributed positively and negatively to the separation on the PC1 axis, respectively (Supplemental Figure S3E).

At the genus level, factor loading for PC2 showed that the relative abundances of the genera *Lachnospiraceae NK4A136* group and *Alistipes* contributed positively and negatively to the separation on the PC2 axis, respectively (Figure 3E). On the other hand, in the CFA-injected group, neither PC1 nor PC2 values had statistical differences between the ileum and feces at the phylum (PC1, *p* = 0.10; and PC2, *p* = 0.37) and genus (PC1, *p* = 0.20; and PC2, *p* = 0.11) levels (Supplemental Figure S3D, Figure 3D).

### 2.4. Alterations of the Microbiota Compositions in the Ileum and Feces

**Figure 4:**
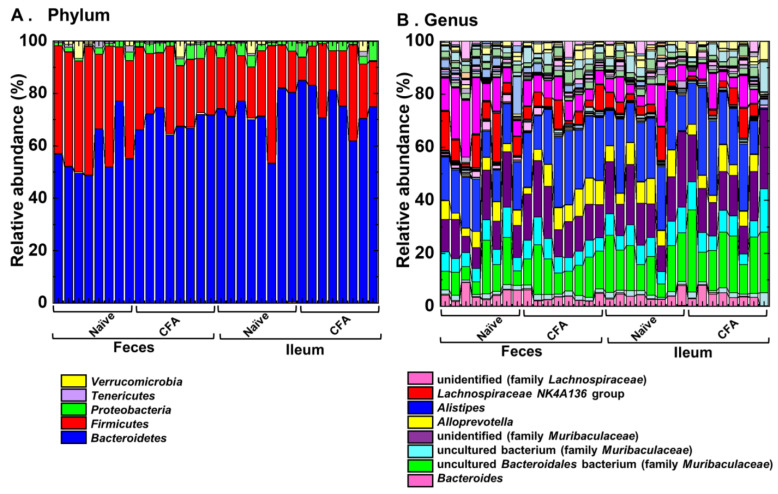
Relative abundances of ileal and fecal bacteria. Using 16S rRNA sequencing, we analyzed the relative abundances of individual bacteria at the phylum (**A**) and genus levels (**B**). Each group was composed of eight mice.

To investigate whether adjuvant injections could affect the relative abundances of individual bacterial phylum and genus, we compared them at the phylum and genus levels between the naïve and CFA-injected groups (Figure 4, Table 2 and Table 3). We found significant compositional differences in the microbiota between the naïve and CFA-injected groups at the phylum and genus levels in both the ileal and fecal samples (Figure 4A,B). In the CFA-injected group, at the phylum level, we found increased abundances of *Bacteroidetes* and *Proteobacteria* and decreased abundance of *Firmicutes* in feces, as well as the decreased abundance of *Cyanobacteria* in the ileum (Table 2). At the genus level, we found increased abundances of two genera (*Clostridium sensu stricto 1* and *Alistipes*) and decreased abundances of 14 genera in feces, as well as increased abundances of four genera and decreased abundances of three genera in the ileum (Table 3). On the other hand, there were no common genera which showed different abundances between naïve and CFA-injected mice in the ileum and feces.

We also compared the relative abundances of individual bacterial phylum and genus between the ileum and feces in the naïve and CFA-injected groups. We found significant differences in the bacterial abundances between the ileum and feces in the naïve group: two of total 13 taxa at the phylum level, and 18 of total 159 taxa at the genus level; in the CFA-injected group: one taxon at the phylum level, and 10 taxa at the genus level (Supplemental Tables S1 and S2). We found four common genera (*Gordonibacter*, *ASF356*, *Lachnoclostridium*, and *Ruminococcaceae UCG-010*) that were decreased in feces in the CFA-injected group, which were also decreased in the ileum compared with feces in the naïve group (Table 3, Supplemental Table S2).

### 2.5. Gut Microbiota Associates with Antibody Isotypes and Antimycobacterial Antibody Responses

**Figure 5:**
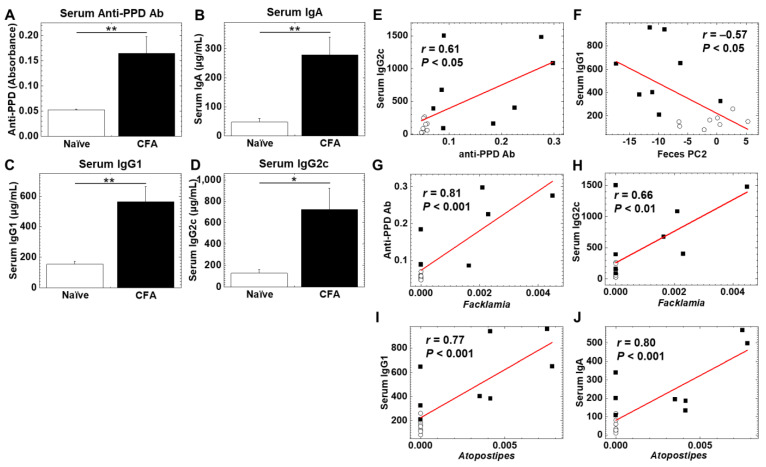
(**A**–**D**) Levels of serum antibody (Ab) against purified protein derivative (PPD) of *Mycobacterium tuberculosis*, a component of CFA, and immunoglobulin isotypes (IgA, IgG1, and IgG2c) in the CFA-treated and naïve groups. Serum anti-PPD Ab (**A**), IgA (**B**), IgG1 (**C**), and IgG2c (**D**) were quantified by enzyme-linked immunosorbent assays (ELISAs). In the CFA-injected group, levels of anti-PPD Ab, IgA, IgG1, and IgG2C were higher than the naïve group (*, *p* < 0.05; **, *p* < 0.01 Student’s *t* test). Values are the mean + standard error (SE). (**E**,**F**) Correlation analyses between serum IgG2c and anti-PPD Ab (**E**), and serum IgG1 and fecal PC2 values (**F**) (○, naïve; ■, CFA). (**G**–**J**) Pattern matching between Ab levels and relative abundance of ileal bacteria: anti-PPD Ab and the genus *Facklamia* (**G**), serum IgG2c and the genus *Facklamia* (**H**), serum IgG1 and the genus *Atopostipes* (**I**), serum IgA and the genus *Atopostipes* (**J**) (○, naïve; ■, CFA).

To determine whether adjuvant injections could alter humoral immune responses, we quantified serum antibody isotypes (IgA, IgG1, and IgG2c) and antibody against purified protein derivatives (PPD) of *M. tuberculosis*, a component of CFA, using enzyme-linked immunosorbent assays (ELISAs) (Figure 5A–D). We found significantly higher levels of anti-PPD antibody, IgA, IgG1, and IgG2c in the CFA-injected group than in the naïve group (*p* < 0.05, Student’s *t* test). We conducted correlation analyses among the levels of anti-PPD antibody, IgA, IgG1, IgG2c, as well as PC1 and PC2 values (reflecting the overall microbiome profiles, shown in Figure 2) of the fecal microbiome PCA. We found that there were moderate correlations between anti-PPD antibody versus the IgG2c isotype, and PC2 values of feces versus the IgG1 isotype with statistical differences (*p* < 0.05, Student’s *t* test, Figure 5E,F). Thus, serum anti-mycobacterial humoral immune response was related to Th1-associated IgG2c production; the overall fecal microbiome profiles were related to Th2-associated IgG1 production.

We also conducted pattern matching between the antibody levels and relative abundances of individual bacteria at the genus level to determine whether individual bacterial abundances were correlated with the antibody levels (Table 4, Table 5, Table 6 and Table 7). Based on the correlation coefficients [42], the relative abundances of 15 genera (eight genera in feces and seven genera in the ileum) were correlated with anti-PPD antibody levels (Table 4). Among these genera, two genera were significantly increased in the ileum, and three genera were decreased in feces of the CFA-injected group, compared with the naïve group (Table 3). Although eight genera (four genera in feces and four genera in the ileum) were correlated with the IgA concentrations, only the genus *Atopostipes* in the ileum was significantly increased in the CFA-injected group (Table 5). Among 23 genera (15 genera in feces and eight genera in the ileum) correlated with the IgG1 concentrations (Table 6), the genus *Atopostipes* in the ileum was significantly increased, and six genera in feces were decreased in the CFA-injected group. Among six genera (one genus in feces and five genera in the ileum) correlated with the IgG2c concentrations (Table 7), only the genus *Facklamia* in the ileum was significantly increased in the CFA-injected group. Thus, among the bacterial genera positively correlated with anti-PPD and antibody isotype responses, only three genera, including *Facklamia*, an unidentified genus in the family *Burkholderiaceae*, and *Atopostipes*, in the ileum were increased in the CFA-injected group; *Facklamia* and the unidentified genus in the family *Burkholderiaceae* were related with anti-PPD and IgG2c levels, and *Atopostipes* were related with IgA and IgG1 levels (Figure 5G–J). On the other hand, among ten bacterial genera in feces negatively correlated with anti-PPD antibody (three genera in Table 4) or IgG1 (seven genera in Table 6) levels, all bacterial genera except *Dorea* were significantly decreased in the CFA-injected group. Here, adjuvant injections resulted in the production of high levels of anti-PPD and antibody isotype responses, each antibody response of which seemed to change the relative abundances of distinct bacterial genera.

Lastly, we conducted PCA using PC1 and PC2 values in the PCA of the microbiome data, and the antibody level data from the naïve and CFA-injected groups (Supplemental Figure S4). PCA separated the CFA-injected group from the naïve group by PC1 values (*p* < 0.01, Student’s *t* test). PC1 and PC2 explained 41.8% and 18.2% of the variation in the PCA, respectively. Factor loading for PC1 showed that all three antibody isotypes (IgA, IgG1, and IgG2c) positively contributed to the separation on the PC1 axis.

## 3. Discussion

Previously, treatment with several agents, including antibiotics and probiotics, has been shown to alter the gut bacterial compositions [28,43,44]. In this study, we demonstrated that adjuvant injections could also alter the gut microbiota, particularly the bacterial compositions in feces, rather than those in the ileum. First, we demonstrated that the CFA-injected group had decreased alpha diversities in feces, but not in the ileum (Figure 1). Generally, in humans, reduced microbial diversities have been considered as an indicator of disease conditions [31,34]. However, in autoimmune animal models, including EAM [18] and EAU [25], the alpha diversities were increased; in EAU, antibiotics treatment reversed the diversity and suppressed disease activity [25]. On the other hand, in human MS and its autoimmune animal models, EAE, the changes in alpha diversities were inconsistent among the reports [31]. Thus, the effects of alpha diversities in the fecal microbiota on disease activities differed among the disease conditions.

The bacterial compositions and diversities have been shown to differ between the ileum and feces. For example, Gu et al. showed the highest diversities in the cecum, colon, and feces, and the lowest diversities in the jejunum and ileum along the mouse gastrointestinal tract [41]. In this study, we found higher alpha diversities in feces than in the ileum in the naïve group, but not in the CFA-injected group (Supplemental Figure S1). Similarly, when we compared the numbers of bacterial phyla and genera having statistical differences in the bacterial abundances between the ileum and feces, these numbers were higher in the naïve group than in the CFA-injected group (Supplemental Tables S1 and S2). Thus, adjuvant injections seemed to decrease the bacterial diversities between the ileum and feces.

Using PCA, we compared the overall microbiota profiles between the naïve and CFA-injected groups and found that they differed in feces, but not in the ileum (Figure 2). We also found that, although the overall microbiota profiles of the ileum and feces significantly differed in the naïve group, the CFA-injected group had no differences in the microbiota profiles between the two anatomical sites; this can be explained by alterations of the fecal microbiota after CFA injection (Figure 3). The different influence of CFA injection on the ileal versus fecal microbiota was likely due to the injection site of CFA, i.e., the tail base, whose regional lymph nodes are the inguinal lymph nodes. We harvested the ileal bacteria from ileal contents, and the fecal bacteria from the rectum and anal canal [45]. Here, the tail base and feces had the common lymphatic drainage system, i.e., the inguinal lymph nodes; the draining lymph nodes of the ileum are the mesenteric lymph node nodes. As a result of the different lymphatic drainage pathways between the ileum and feces, CFA injection could only affect the fecal microbiota in our study. The different effects of adjuvant injections on the ileal versus fecal microbiota may also be due to the composition differences of commensal bacteria in the ileum and feces [41], which can be affected differently by anti-PPD and antibody isotype responses induced by adjuvant injections.

At the genus level, we found that, in feces but not in the ileum, the relative abundances of the genera *Lachnospiraceae NK4A136* group and *Alistipes* were positively and negatively correlated with PC2 values, respectively, in the naive (Figure 3E). The genus *Lachnospiraceae NK4A136* group belongs to the phylum *Firmicutes*, family *Lachnospiraceae*, and its decreased relative abundance has been reported in Alzheimer’s disease [46]. The genus *Alistipes* belongs to the phylum *Bacteroidetes*, family *Rikenellaceae*, and has been reported to play pathogenic roles in human colorectal cancer, anxiety, and depression, although it could play protective roles in human cardiovascular disease, colitis, and autism [47].

In Figure 4, we compared the relative abundances of individual bacteria between the naïve and CFA-injected groups. In the CFA-injected group, we found increased abundances of two genera and decreased abundances of 14 genera in feces, and increased abundances of four genera, and decreased abundances of three genera in the ileum (Table 3). Previously, Johanson II et al. compared the fecal microbiota between naïve and CFA-injected groups; the CFA injection group had decreased levels of several genera, including *Anaerostipes* and *Stomatobaculum*, and increased levels of certain genera, including *Akkermansia* and *Peptococcus−rc4−4*, without changes in alpha diversities (shown in Figure S2E of [15]). When we compared the fecal microbiota changes of the CFA-injected group between our current (Table 3) versus Johanson II’s studies, the relative abundances of four bacterial genera (*Clostridium sensu stricto 1*, *Lachnoclostridium*, *Lachnospiraceae FCS020 group*, and *Ruminococcaceae UCG-010*) were altered in both studies. Although the two studies had discrepancies in the relative abundances of other bacteria, this could be due to differences in environmental conditions in the animal housing facilities, different vendors, and some other factors, as reported previously [48]. Nevertheless, the two studies demonstrated that the CFA-injected group had significant changes in the fecal microbiota. It should be noted, however, that the CFA-injected group in our current study received an additional adjuvant PT, which has been used in the induction of autoimmune models [49]. Here, injection of PT may also influence the gut microbiota [50].

Since CFA contains the components of *M. tuberculosis*, we examined whether anti-mycobacterial antibody responses (i.e., anti-PPD antibody levels) could be associated with the gut microbiota (Figure 5G–J). We found that anti-PPD responses were positively correlated with the five genera in feces and seven genera in the ileum; among these bacterial genera, in the ileum two genera were increased significantly in the CFA-injected group. Although anti-PPD antibody responses were negatively correlated with three genera in feces only, all three genera were significantly decreased in the CFA-injected group (Table 4). These results suggest that anti-adjuvant immune responses may affect distinct individual bacterial genera in the ileum and feces. In humans, induction of antimycobacterial immunity by the bacillus Calmette–Guérin (BCG) vaccine was associated positively with the genera *Ruminococcus* and *Eggerthella lenta* in the gut microbiota [51]. In infants following BCG, polio, tetanus toxoid, and hepatitis B virus vaccinations, adaptive immune responses to the vaccines were positively and negatively correlated with *Actinobacteria* and *Enterobacter*, respectively, in the gut microbiota [52]. On the other hand, in pulmonary tuberculosis patients, the gut microbiota was characterized by increased genera *Prevotella* and *Enterococcus* and decreased genera *Fecalibacterium*, *Bacteroides*, *Ruminococcus*, and *Dorea* [53]. In another pulmonary tuberculosis study, the genera *Prevotella* and *Lachnospira* were decreased in patients, compared with healthy controls [54]. Among these gut bacterial changes reported in human studies, we only found that the genus *Ruminococcus* was decreased in the CFA-injected group in the ileum and feces; the genus *Ruminococcus* has been reported to be protective in atopic dermatitis [55]. Although both anti-PPD antibody and BCG vaccine have antimycobacterial functions, we found differences in the gut bacterial changes by CFA and BCG injections. This may be due to differences in the microbiota compositions between mice and humans [56]. On the other hand, since the gut microbiota has been suggested to act as a natural adjuvant to enhance immune responses to vaccines [57], the presence of certain gut bacteria can be not only the results of antimycobacterial immunization, but also the cause (or adjuvant) of induction of anti-mycobacterial immunity.

In our study, the CFA-injected group had higher serum IgA, IgG1, and IgG2c concentrations, compared with the naïve group (Figure 5A–D). Using pattern matching, we found eight genera correlated with the IgA concentrations (Table 5), 23 genera correlated with the IgG1 concentrations (Table 6), and six genera correlated with the IgG2c concentrations (Table 7). Among these genera positively correlated with anti-PPD or antibody isotype responses, only three genera, including *Facklamia* and *Atopostipes*, in the ileum were increased in the CFA-injected group. The genus *Facklamia* in the ileum was strongly correlated with not only the IgG2c concentrations but also the serum anti-PPD antibody levels (Figure 5G,H); the genus *Facklamia* are Gram-positive bacteria and has been associated with invasive diseases, including meningitis [58]. The genus *Atopostipes* in the ileum was strongly correlated with the serum IgG1 and IgA concentrations (Figure 5I,J); the genus *Atopostipes* are Gram-positive bacteria isolated from a swine manure storage pit [59].

Among 10 bacterial genera in feces negatively correlated with anti-PPD antibody (three genera in Table 4) or IgG1 (seven genera in Table 6) levels, nine genera, including *Rikinella* [60], *ASF356*, *Gordonibacter* [61], and *Anaerovorax,* were significantly decreased in the CFA-injected group. On the other hand, we demonstrated that IgG1 concentrations were significantly correlated with PC2 values of fecal microbiome PCA (Figure 5F), suggesting that the altered overall fecal microbiota profiles in the CFA-injected group could be associated with IgG1 responses. In addition, when we conducted PCA of the ileal and fecal microbiome and all antibody data, we found that all three antibody isotypes (IgA, IgG1, and IgG2c) contributed to the differences between the naïve and CFA-injected groups (Supplemental Figure S4, and Table S3). Thus, adjuvant injections resulted in higher anti-PPD production and antibody isotype responses, each antibody response of which could affect not only the relative abundances of distinct bacterial genera, but also the overall fecal microbiome profiles.

In the current study, we have used CFA and PT as adjuvants. CFA contains several potential immunomodulatory molecules [5], including muramyl peptide, mycolic acid, lipoarabinomannan, heat shock proteins, and unmethylated DNA. Among the mycobacterial components in CFA, muramyl peptide, mycolic acid, and lipoarabinomannan, have been shown to enhance Th1 immune responses; PT has also been shown to enhance Th1 immune responses. Thus, the individual effects of immunomodulatory molecules contained in adjuvants were not distinguishable. Nevertheless, clarifying the precise effect on the microbiota by each immunomodulatory molecule will help to establish more efficient modulation of the gut microbiota, leading to better induction of autoimmune models in the future.

In conclusion, we demonstrated that adjuvant injections alone could alter diversities and compositions of the gut microbiota, particularly in feces, and that alterations of the microbiota were correlated with anti-mycobacterial and immunoglobulin isotype antibody responses. Therefore, the gut microbiota in CFA-induced autoimmune models could be influenced by adjuvant itself, to some extent. In this study, although we examined the effect of adjuvants on bacteria in ileal contents and feces, bacteria have also been shown to exist on the mucosal surface [16] and in the intestinal lymphoid tissue (lymphoid-tissue-resident commensal bacteria, LRCs) [62], and can play immunomodulatory roles. Future analyses of mucosal bacteria and LRCs as well as mycobiome and virome in the gut may clarify more precise effects of adjuvant injections on the gut microbiota.

## 4. Materials and Methods

### 4.1. Animal Experiments

We purchased six-week-old female C57BL/6 mice from CLEA Japan, Inc. (Tokyo, Japan). We maintained mice under specific-pathogen-free conditions in our animal care facility at Kindai University Faculty of Medicine (Osakasayama, Osaka, Japan). All experimental procedures were approved by the Institutional Animal Care and Use Committee of Kindai University Faculty of Medicine and performed according to the criteria outlined by the National Institutes of Health (NIH) [63].

We divided mice into two groups: the naïve and CFA-injected groups (eight mice per group). For the CFA-injected group, we sensitized mice subcutaneously with CFA that consisted of IFA (BD, Franklin Lakes, NJ, USA) and *M. tuberculosis* H37 Ra (BD) on days 0 and 19. The final concentration of *M. tuberculosis* in the CFA emulsions was 2 mg/mL (400 μg/mouse). We also injected CFA-injected mice with 300 ng of PT (List Biological Laboratories, Campbell, CA, USA) intraperitoneally on days 0 and 2. PT has been used by other research groups, including Johanson II et al. [25], for CFA-induced autoimmune models [64]. We monitored their body weight changes and any clinical signs daily for 5 weeks. After 5 weeks, we collected the blood samples for ELISAs, perfused mice with phosphate-buffered saline (PBS), and harvested feces from the rectum and anal canal for microbiome analyses. We also collected ileal contents from the ileum by flushing with distilled water. All samples were frozen in liquid nitrogen and stored at −80 °C until examined [65].

### 4.2. Antibody ELISA

Sera were obtained from the blood samples by centrifugation at 2775× *g* at 4 °C for 20 min. We coated 96-well flat-bottom Nunc-Immuno plates (Thermo Fisher Scientific, Inc., Waltham, MA, USA) with 10 μg/mL of PPD (National Institute for Biological Standards and Control, Hertfordshire, UK) [66] or goat anti-mouse IgA, IgG1, or IgG2c capture antibody (SouthernBiotech, Birmingham, LA, USA). Sera were diluted at 2^7^ for anti-PPD, and 10^3^ to 10^7^ for IgA, IgG1, and IgG2c, and added to the plates followed by a peroxidase-conjugated anti-mouse total IgG (H+L) (2000-fold dilution, Thermo Fisher Scientific, Inc.) for anti-PPD antibody and peroxidase-conjugated anti-mouse IgG F(ab’)_2_ (5000-fold dilution, Jackson ImmunoResearch Laboratories, Inc., West Grove, PA, USA) for IgA, IgG1, and IgG2c. Immunoreactive complexes were detected with 3,3’,5,5’-tetramethylbenzidine (TMB) (BD). The absorbances were measured at 450 nm, using the Synergy H1 Hybrid Multi-Mode Microplate Reader (Agilent Technologies, Inc., Santa Clara, CA, USA). We conducted ELISAs in duplicate wells of 96-well plates.

### 4.3. 16S rRNA Amplicon Sequencing

We extracted DNA from ileal contents and feces using the QIAamp^®^ Fast DNA Stool Mini Kit (Qiagen, Germantown, MD, USA), according to the manufacturer’s instruction [67]. The 16S rRNA amplicon sequencing was conducted on MiSeq (Illumina, San Diego, CA, USA) by MR DNA (Shallowater, TX, USA, http://www.mrdnalab.com/bioinformatics.html, accessed on 27 January 2020). Fastq data were demultiplexed, denoised, aligned to the bacterial rRNA database in SILVA (https://www.arb-silva.de/, accessed on 2 June 2020), and visualized by QIIME 2 [68]. Fastq files and processed data were deposited to the Sequence Read Archive (SRA) in the National Center for Biotechnology Information (NCBI) (Accession number, PRJNA914988; URL, https://www.ncbi.nlm.nih.gov/bioproject/PRJNA914988, accessed on 1 February 2023).

### 4.4. Bioinformatics Analyses

#### 4.4.1. Alpha Diversity

We conducted alpha diversity analyses of the microbiome between the naïve and CFA-injected groups and between the ileal and fecal samples, using QIIME 2 [68]. We used the Faith’s phylogenetic diversity index for bacterial richness, Pielou’s evenness index for bacterial evenness, and Shannon index for the combination of both [67].

#### 4.4.2. PCA

To determine the variation of the overall microbiome profiles of the ileal and fecal microbiome data, we conducted PCA, using an R program “prcomp,” as described previously [69]. Factor loading for the PC1 or PC2 was used to rank the bacterial genera or phyla which contribute to the distributions of the samples on the PC1 or PC2 axis. We plotted a graph of PCA with ellipses of an 80% confidence interval, using R packages, “dplyr” and “ggplot2.”

#### 4.4.3. Pattern Matching

To examine the associations between the antibody (anti-PPD, IgA, IgG1, IgG2c) levels and microbiome data, we conducted pattern matching, using R. We compared each antibody level versus the relative abundances of bacteria at the genus level [70]. The values more than 0.7 or less than −0.7 in the Spearman’s rank correlation coefficient (*r*) with *p* < 0.05 (calculated by Microsoft Excel, Microsoft Corporation, Redmond, WA, USA) were considered as a highly positive or negative correlation, respectively [42]. When the *r* value is from 0.5 to 0.7 or from −0.5 to −0.7 with *p* < 0.05, it was considered as a moderate correlation [42].

### 4.5. Statistical Analyses

Using the OriginPro 2022 (OriginLab Corporation, Northampton, MA, USA), we performed the Student’s *t* test for parametric data. *p* < 0.05 was considered as a statistically significant difference [71].

## Figures and Tables

**Table 1:** Microbiota studies in CFA-induced autoimmune animal models.

Human Disease	Animal Model	Antigen/ Animal/PT *	Sample	Microbiota Findings	Other Findings	Ref
Guillain–Barré syndrome (GBS)	Experimental autoimmune neuritis (EAN)	myelin P2 protein/ Lewis rats/ PT	feces	↑: *Akkermansia*, *Escherichia*, and *Coprococcus*; ↓: *Lactobacillus*, *Ruminococcus*, and *Clostridium*	Treatment with *Bifidobacterium* suppressed EAN	[14]
Multiple sclerosis (MS)	Experimental autoimmune encephalomyelitis (EAE)	MOG/ C57BL/6 mice/ PT	feces	↑: *Clostridiaceae*, *Ruminococcaceae*, and *Peptostreptococcaceae* ↓: *Lactobacillaceae* ↓: alpha diversity (EAE vs. CFA-injected groups)	CFA injection alone altered microbiota ↑: *Anaerostipes, Stomatobaculum,* ↓: *Akkermansia, Peptococcus−rc4−4*	[15]
		MOG/ C57BL/6 mice/ PT	feces, ileal content, ileal mucosa	↑: *Ruminococcus bromii* and *Blautia* *(Ruminococcus) gnavus* in feces ↑: *Turicibacter sp.* and *Alistipes finegoldii* in ileal contents ↑: *Burkholderia spp.* and *Azoarcus spp.* in ileal mucosa	Ileal, but not fecal, microbiota associated with EAE severity	[16]
Myasthenia gravis (MG)	Experimental Autoimmune myasthenia gravis (EAMG)	TAChR/ Lewis rats	feces	↑: *Ruminococcaceae*/*Lachnospiraceae* ratio ↓: *Tenericutes*/*Verrucomicrobia* ratio	Treatment with *Bifidobacterium* suppressed EAMG	[17]
Myocarditis	Experimental autoimmune myocarditis (EAM)	MyHC-α/ BALB/c mice	feces	↑: *Firmicutes*/*Bacteroidetes* ratio ↑: alpha diversity (EAM vs. saline-injected groups)	Treatment with FMT suppressed EAM without change in diversity	[18]
		MyHC-α/ BALB/c mice/ PT	feces	↑: *Firmicutes* ↓: *Bacteroidetes*, *Proteobacteria*	Treatment with antibiotics suppressed EAM pathology	[19]
Rheumatoid arthritis (RA)	Collagen-induced arthritis (CIA)	type II collagen /SD rats	feces	↑: *Proteobacteria*, *Actinobacteria* ↓: *Tenericutes*	Treatment with total glucosides of paeony (TGP) suppressed CIA and restored gut dysbiosis	[20]
		type II collagen /ICR mice	feces	↑: *Firmicutes, Bacteroidetes* ↓: *Proteobacteria, Actinobacteria* alpha diversity: Chao1↑, Shannon ↓ (CIA vs. saline-injected groups)	Treatment with tuna oil suppressed CIA and restored gut dysbiosis	[21]
	Adjuvant-induced arthritis (AIA)	*Mycobacterium tuberculosis* suspension/ SD rats	feces	↑: *Candidatus Arthromitus sp. SFB-rat Yit* and *Klebsiella pneumoniae* ↓: *Lactobacillus hominis*, *L. reuteri*, and *L. vaginalis*	Treatment with *Lactobacillus* *casei* suppressed AIA and restored gut dysbiosis	[22]
Thyroiditis	Experimental autoimmune thyroiditis (EAT)	thyroglobulin/ CBA/CaH mice	–	microbiota was not examined	Probiotic treatment with *Lactobacillus rhamnosus* HN001 and *Bifidobacterium lactis* HN019 had no effect on EAT	[24]
Uveoretinitis	Experimental autoimmune uveoretinitis (EAU)	IRBP/ B10.RIII mice	cecal content	↑: *Anaeroplasma, Turicibacter* and *Oscillospora* ↓: *Desulfovibrio*, *Clostridium*, *Staphylococcus*, *Adlecreutzia*, and *Lactobacillus* ↓: alpha diversity (antibiotics-treated vs. untreated EAU groups)	Treatment with antibiotics suppressed EAU	[25]

*, pertussis toxin (PT) was injected as an additional adjuvant. Abbreviations: CFA, complete Freund’s adjuvant; FMT, fecal microbiota transplantation; IRBP, interphotoreceptor retinoid binding protein; MOG, myelin oligodendrocyte glycoprotein; MyHC-α, cardiac α-myosin heavy chain peptide; TAChR, *Torpedo californica* acetylcholine receptor. ↑, increased compared with controls; ↓, decreased compared with controls.

**Table 2:** Bacterial phylum changes in the CFA-injected group compared with the naïve group.

Change	Feces	Ileum
↑	*Bacteroides*, *Proteobacteria*	–
↓	*Firmicutes*	*Cyanobacteria*

↑, Significant increase compared with the naïve group (*p* < 0.05, Student’s *t* test). ↓, Significant decrease compared with the naïve group (*p* < 0.05, Student’s *t* test). –, No differences compared with the naïve group.

**Table 3:** Bacterial genus changes in the CFA-injected group compared with the naïve group.

Change	Feces	Ileum
↑	*Clostridium sensu stricto 1* *Alistipes*	*Eubacterium coprostanoligenes* group *Facklamia* Family *Burkholderiaceae*; genus unidentified *Atopostipes*
↓	Family *Christensenellaceae*; genus uncultured *Gordonibacter* Family *Erysipelotrichaceae*; genus uncultured bacterium *Anaerovorax* *Eubacterium xylanophilum* group *ASF356* *Lachnoclostridium* *Lachnospiraceae FCS020* group Family *Lachnospiraceae*; genus unidentified *Peptococcus* Family *Peptococcaceae*; genus uncultured *Rikenella* *Ruminococcaceae UCG-009* *Ruminococcaceae UCG-010*	Family XIII; genus unidentified *Ruminococcaceae UCG-004* *Ruminococcus 1*

↑, Significant increase compared with the naïve group (*p* < 0.05, Student’s *t* test). ↓, Significant decrease compared with the naïve group (*p* < 0.05, Student’s *t* test).

**Table 4:** Bacterial genera correlated with anti-PPD antibody.

Correlation	Feces		Ileum	
	Genus	*r* *	Genus	r*
Positive (*r* ≥ 0.5)	*Cloacibacterium* *Alicyclobacillus* Family *Burkholderiaceae*; genus unidentified *Acinetobacter* *Rhodanobacter*	0.59 0.58 0.58 0.58 0.58	*Facklamia* ** Family *Burkholderiaceae*; genus unidentified ** Sporosarcina Family *Desulfarculaceae*; genus uncultured Family *Erysipelotrichaceae*; genus uncultured Family *Erysipelotrichaceae*; genus unidentified Family *Prevotellaceae*; genus unidentified	0.82 0.67 0.60 0.59 0.58 0.54 0.52
Negative (*r* ≤ −0.5)	*Eubacterium xylanophilum* group *** Family *Christensenellaceae*; genus uncultured *** Lachnoclostridium ***	−0.64 −0.56 −0.54	–	

* *r*; correlation coefficient; **; significantly increased genus in the CFA-injected group, compared with the naïve group; ***; significantly decreased genus in the CFA-injected group, compared with the naïve group.

**Table 5:** Bacterial genera correlated with serum IgA.

Correlation	Feces		Ileum	
	Genus	*r* *	Genus	*r* *
Positive (*r* ≥ 0.5)	*Ruminococcus 1* *Micrococcus* Family *Saprospiraceae*; genus unidentified *Turicibacter*	0.53 0.53 0.53 0.51	*Atopostipes* ** Family *Bacillaceae*; genus unidentified *Dorea* *Faecalibaculum*	0.81 0.64 0.55 0.52
Negative (*r* ≤ −0.5)	–		–	

* *r*; correlation coefficient; **; significantly increased genus in the CFA-injected group, compared with the naïve group.

**Table 6:** Bacterial genera correlated with serum IgG1.

Correlation	Feces		Ileum	
	Genus	*r* *	Genus	*r* *
Positive (*r* ≥ 0.5)	*Turicibacter* *Ruminococcaceae UCG-004* *Paenarthrobacter* uncultured *Sphingobacteriales* bacterium *Karenia brevis* Family *Cellvibrionaceae*; genus unidentified *Enhydrobacter* *Salinispira*	0.59 0.56 0.54 0.54 0.54 0.54 0.54 0.54	*Atopostipe s* ** Family *Erysipelotrichaceae*; genus unidentified *Hydrogenoanaerobacterium* *Candidatus Soleaferrea* *Ruminococcaceae NK4A214* group *Dorea* *Lachnospiraceae UCG-001* *Psychrobacter*	0.78 0.71 0.64 0.61 0.57 0.55 0.55 0.54
Negative (*r* ≤ −0.5)	*Rikenella* *** ASF356 *** Gordonibacter *** Dorea Family *Christensenellaceae*; genus uncultured *** Family *Peptococcaceae*; genus uncultured *** Anaerovorax ***	−0.52 −0.52 −0.52 −0.54 −0.55 −0.56 −0.56	–	

* *r*; correlation coefficient, **; significantly increased genus in the CFA-injected group, compared with the naïve group, ***; significantly decreased genus in the CFA-injected group, compared with the naïve group.

**Table 7:** Bacterial genera correlated with serum IgG2c.

Correlation	Feces		Ileum	
	Genus	*r* *	Genus	*r* *
Positive (*r* ≥ 0.5)	*Dubosiella*	0.52	*Facklamia* ** Hydrogenoanaerobacterium Family *Bacillaceae*; genus unidentified Family *Prevotellaceae*; genus unidentified *Romboutsia*	0.66 0.62 0.58 0.57 0.52
Negative (*r* ≤ −0.5)	–		–	

* *r*; correlation coefficient; **; significantly increased genus in the CFA-injected group, compared with the naïve group.

## Data Availability

The data presented in this study are openly available in NCBI accession number PRJNA914988 (URL: https://www.ncbi.nlm.nih.gov/bioproject/PRJNA914988, accessed on 1 February 2023).

## Supplementary Materials

The following supporting information can be downloaded at: https://www.mdpi.com/article/10.3390/ijms24032818/s1.
