# Supplementary material for: Adjuvant Injections Altered the Ileal and Fecal Microbiota Differently with Changes in Immunoglobulin Isotypes and Antimycobacterial Antibody Responses
Source: Int J Mol Sci. 2023 Feb 1;24(3):2818. doi: 10.3390/ijms24032818 (PMC9917480; doi:10.3390/ijms24032818)
Supplement: Supplementary file 1 [file ijms-24-02818-s001.zip › Supplmental Figures S1-S3, Tables S1-S2 Khadka et al IJMS 2023_V4.pdf]

Supplemental Materials

Adjuvant Injections Altered the Ileal and Fecal Microbiota Differently with Changes in Immunoglobulin Isotypes and Antimycobacterial Antibody Responses

Sundar Khadka[†], Seiichi Omura[†], Fumitaka Sato, Ikuo Tsunoda*

Department of Microbiology, Kindai University Faculty of Medicine, Osaka, Japan

[†]These authors contributed equally to this work.

***Correspondence:**

Ikuo Tsunoda, M.D., Ph.D.

Department of Microbiology, Kindai University Faculty of Medicine

377-2 Ohnohigashi, Osakasayama, Osaka 589-8511 Japan

E-mail: itsunoda@med.kindai.ac.jp

Keywords: adjuvant, animal model, antibody isotype, bioinformatics, experimental autoimmune encephalomyelitis, *Mycobacterium tuberculosis*, pattern matching

Table of contents

Supplemental Figure S1. Alpha diversities of the microbiome between the ileum and feces in the naïve group and the group injected with complete Freund's adjuvant (CFA) 3

Supplemental Figure S2. Principal component analysis (PCA) of the microbiome data at the phylum level between the naïve and CFA-injected groups 4

Supplemental Figure S3. PCA of the microbiome data at the phylum level between the ileum and feces in the naïve and CFA-injected groups 5

Supplemental Figure S4. PCA of antibody levels and microbiome PC values between the naïve and CFA-injected groups 6

Supplemental Table S1. Bacterial phylum abundances in the ileum compared with feces in the naïve and CFA-injected groups 7

Supplemental Table S2. Bacterial genus abundances in the ileum compared with feces in the naïve and CFA-injected groups 8

Supplemental Table S3....Supplemental Excel File

Accession no.: PRJNA914988, URL: https://www.ncbi.nlm.nih.gov/bioproject/PRJNA914988

- Relative abundances of bacteria at the phylum level.. Sheet 1
- Relative abundances of bacteria at the genus level.. Sheet 2
- Fold changes of the taxa at the phylum and genus levels .... Sheet 3
- Antibody levels and bacterial relative abundance data for pattern matching .... Sheet 4
- Antibody levels and microbiota principal component (PC) 1 and PC2 values for PCA .... Sheet 5

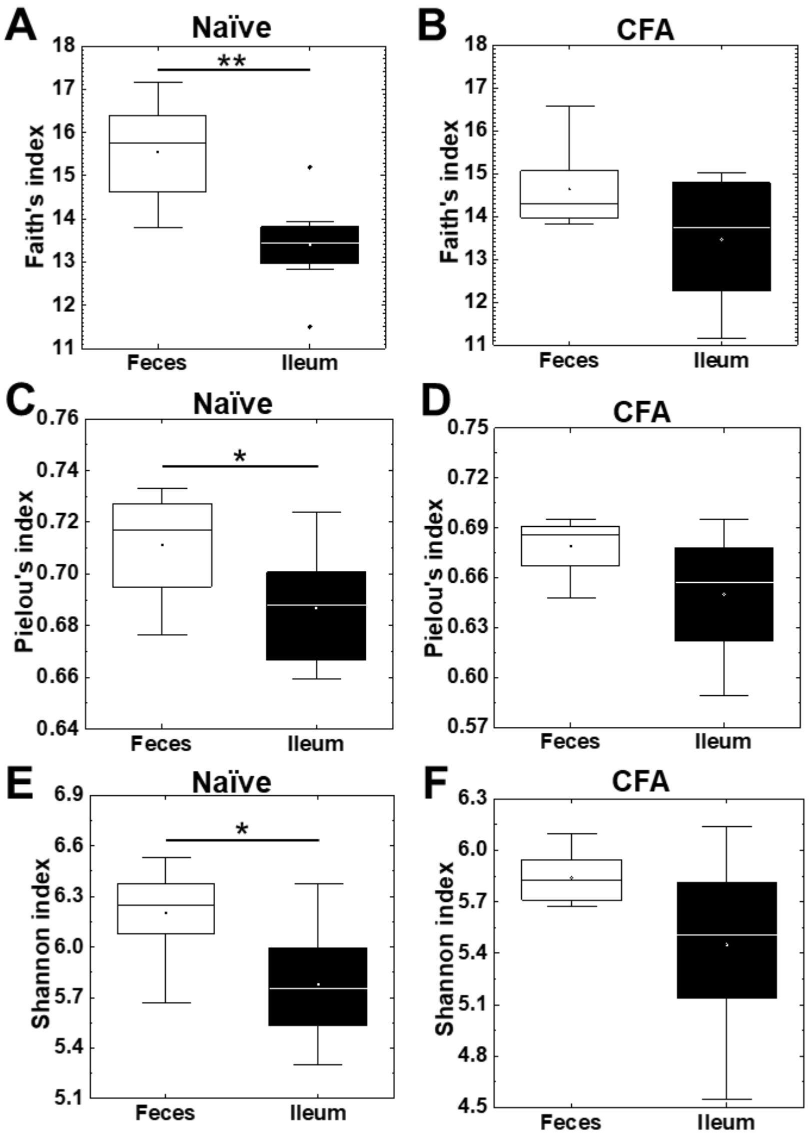

Supplemental Figure S1. Alpha diversities of the microbiome between the ileum and feces in naïve mice and mice injected with complete Freund's adjuvant (CFA). (**A-F**) Using QIIME 2, we compared the richness (number of genera), evenness, and combination of them by the Faith's phylogenetic diversity (**A**, **B**), Pielou's evenness (**C**, **D**), and Shannon (**E**, **F**) indexes, respectively, between the ileum and feces. In the naïve group (**A**, **C**, **E**), all three indexes were significantly different between the ileum and feces, all of which had decreased diversities in the ileum (*, $p < 0.05$; **, $p < 0.01$, Student's t test). In the CFA-injected group (**B**, **D**, **F**), we found that none of them were significantly different between the ileum and feces. In boxplots: the open circle, middle line, box, lower whiskers, upper whiskers, and dots indicate the mean, median, interquartile range, minimum, maximum and outliers, respectively. The total sample number was eight per group.

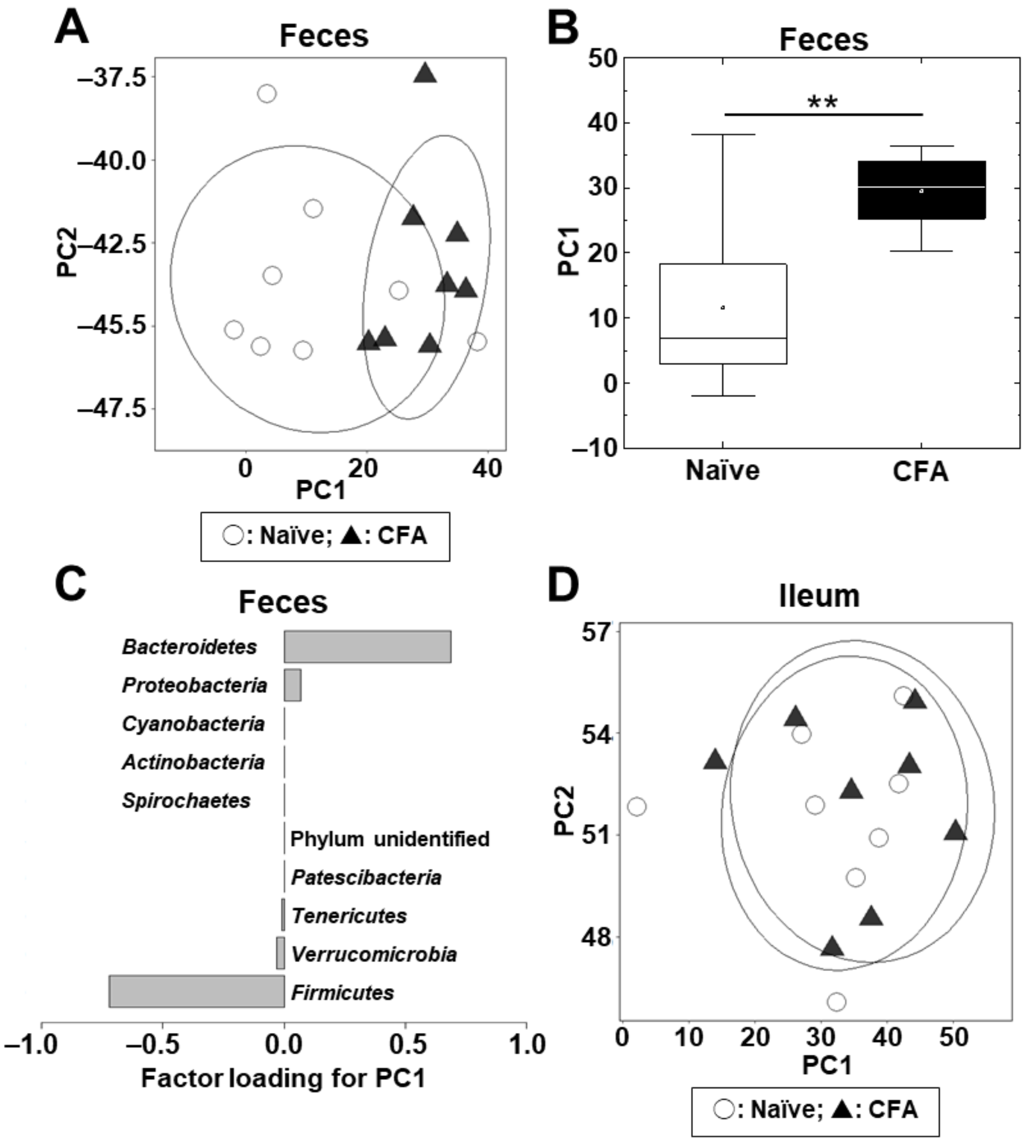

Supplemental Figure S2. Principal component analysis (PCA) of the microbiome data at the phylum level between the naïve (○) and CFA-injected (▲) groups. (**A**, **B**) We conducted PCA, using the fecal samples (**A**), and found that the two groups were separated clearly on principal component (PC) 1 (**, $p < 0.01$, Student's t test) (**B**). Ellipses indicated an 80% confidence interval of each group. (**C**) The phyla *Bacteroidetes* and *Firmicutes* contributed positively and negatively to the PC1 separation, respectively. (**D**) In the ileum, we found that neither PC1 ($p = 0.51$) nor PC2 ($p = 0.77$) separated the two groups. The total sample number was eight per group.

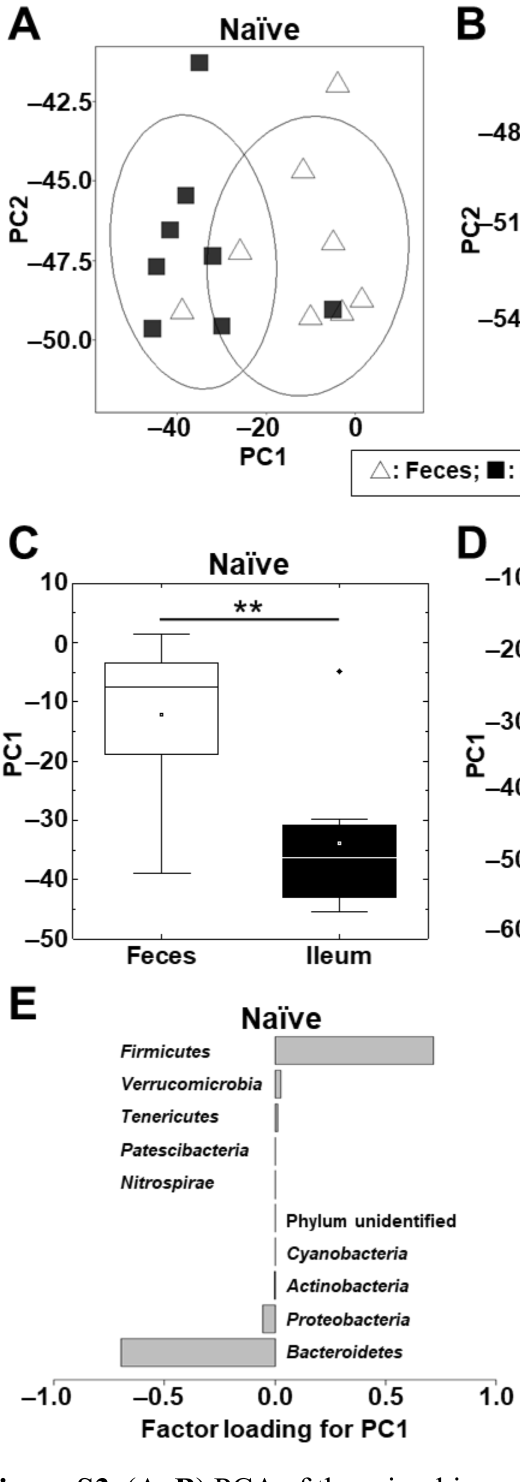

Supplemental Figure S3. (**A**, **B**) PCA of the microbiome data at the phylum level between the ileum (■) and feces (△) in the naïve (**A**) and CFA-injected (**B**) groups. Ellipses indicated an 80% confidence interval of each group. (**C**) In the naïve group, the ileal and fecal samples were separated on PC1 values (**, $p < 0.01$, Student's *t* test). (**D**) In the CFA-injected group, the samples were not separated ($p = 0.10$) between the ileum and feces. (**E**) Factor loading for PC1 showed that the relative abundances of the phyla *Firmicutes* and *Bacteroidetes* contributed positively and negatively to the PC1 distributions, respectively, in the naïve group. The total sample number was eight per group.

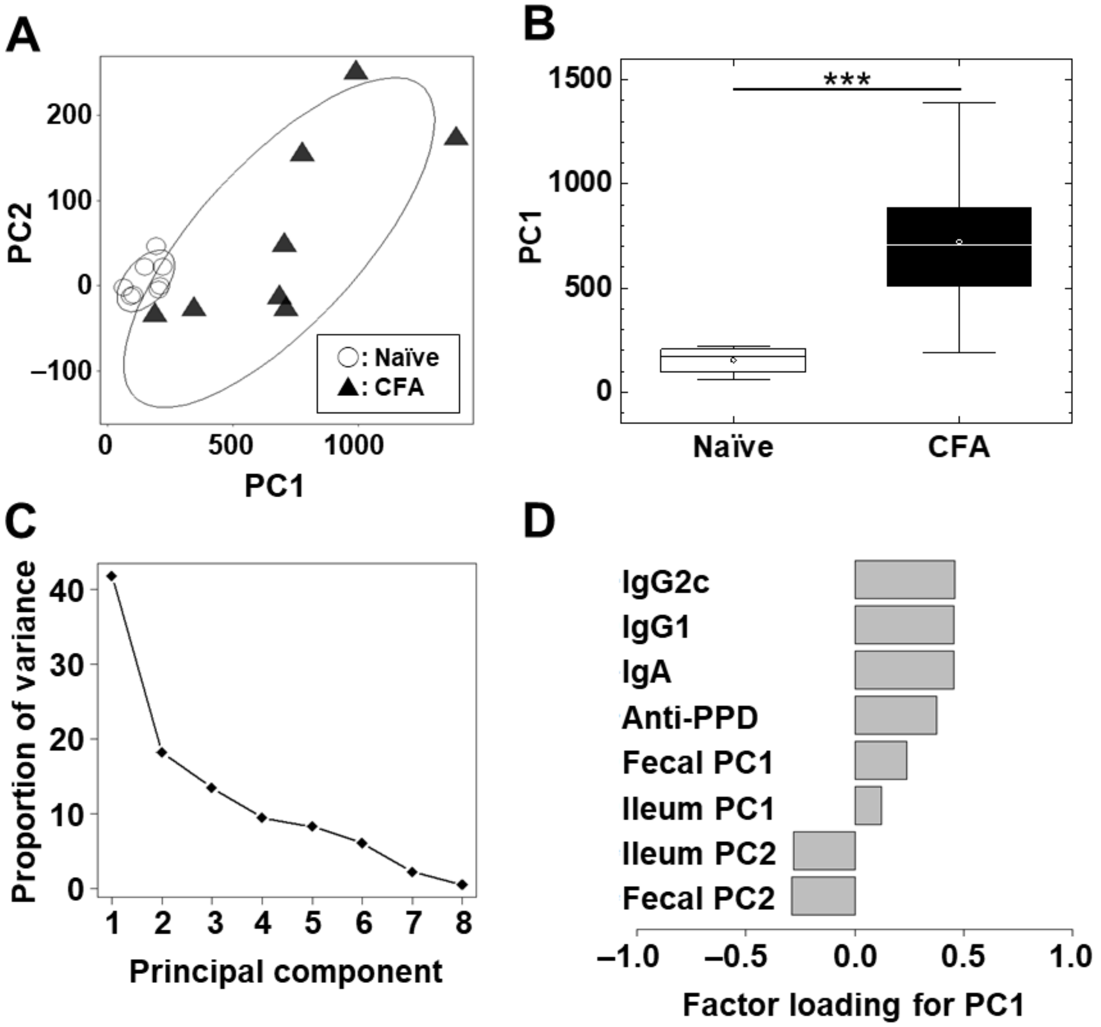

Supplemental Figure S4. (**A**) PCA of antibody levels and microbiome PC values between the naïve (○) and CFA-injected (▲) groups. The ellipses indicated an 80% confidence interval of each group. (**B**) PC1 values were statistically different between the two groups (***, $p < 0.001$, Student's t test). (**C**) PC1 and PC2 explained 41.8% and 18.2% of variation among the samples, respectively. (**D**) Factor loading for PC1 showed that immunoglobulin (IgG2c, IgG1, and IgA) concentrations contributed to the difference between the two groups strongly.

Supplemental Table S1. Bacterial phylum changes in the ileum compared with feces in the naïve and CFA-injected groups

Change	Naïve	CFA
↑	*Bacteroides*	–
↓	*Firmicutes*	*Cyanobacteria*

↑, Significant increase in the ileum compared with feces ($p < 0.05$, Student's *t* test).

↓, Significant decrease in the ileum compared with feces ($p < 0.05$, Student's *t* test).

–, No differences compared with feces.

Supplemental Table S2. Bacterial genus abundances in the ileum compared with feces in the naïve and CFA-injected groups

**Change**	**Naïve**	**CFA**
↑	Family *Clostridiales vadinBB60* group; Genus uncultured *Clostridia* bacterium	*Facklamia*
		Atopostipes
		Corynebacterium 1
		Family *Muribaculaceae*; Genus uncultured *Bacteroidales* bacterium
		Sporosarcina
		Staphylococcus
↓	*Eubacterium nodatum* group	Family *XIII*; genus unidentified
	Family *XIII*; Genus *Family XIII UCG-001*	*Ruminococcaceae UCG-004*
	Family *Christensenellaceae*; Genus uncultured	*Ruminococcaceae UCG-010*
	Gordonibacter	*Ruminococcaceae UCG-014*
	Family *Erysipelotrichaceae*; Genus uncultured bacterium	
	Family *XIII*; Genus *Family XIII AD3011* group	
	Family *Lachnospiraceae*; Genus unidentified	
	Eubacterium xylanophilum group	
	ASF356	
	Dorea	
	Family *Lachnospiraceae*; Genus *GCA-900066575*	
	Lachnoclostridium	
	Lachnospiraceae FCS020 group	
	Lactobacillus	
	Family *Peptococcaceae*; Genus uncultured	
	Ruminiclostridium 5	
	Ruminococcaceae UCG-010	

↑, Significant increase in the ileum compared with feces ($p < 0.05$, Student's t test).

↓, Significant decrease in the ileum compared with feces ($p < 0.05$, Student's t test).
